# Menthol Attenuates Respiratory Irritation and Elevates Blood Cotinine in Cigarette Smoke Exposed Mice

**DOI:** 10.1371/journal.pone.0117128

**Published:** 2015-02-13

**Authors:** Michael A. Ha, Gregory J. Smith, Joseph A. Cichocki, Lu Fan, Yi-Shiuan Liu, Ana I. Caceres, Sven Eric Jordt, John B. Morris

**Affiliations:** 1 Toxicology Program, Department of Pharmaceutical Sciences, School of Pharmacy, University of Connecticut, Storrs, Connecticut, United States of America; 2 Department of Psychiatry, Yale School of Medicine, New Haven, Connecticut, United States of America; 3 Department of Pharmacology, Yale School of Medicine, New Haven, Connecticut, United States of America; 4 Department of Anesthesiology, Duke University School of Medicine, Durham, North Carolina, United States of America; University of South California, UNITED STATES

## Abstract

Addition of menthol to cigarettes may be associated with increased initiation of smoking. The potential mechanisms underlying this association are not known. Menthol, likely due to its effects on cold-sensing peripheral sensory neurons, is known to inhibit the sensation of irritation elicited by respiratory irritants. However, it remains unclear whether menthol modulates cigarette smoke irritancy and nicotine absorption during initial exposures to cigarettes, thereby facilitating smoking initiation. Using plethysmography in a C57Bl/6J mouse model, we examined the effects of L-menthol, the menthol isomer added to cigarettes, on the respiratory sensory irritation response to primary smoke irritants (acrolein and cyclohexanone) and smoke of Kentucky reference 2R4 cigarettes. We also studied L-menthol’s effect on blood levels of the nicotine metabolite, cotinine, immediately after exposure to cigarette smoke. L-menthol suppressed the irritation response to acrolein with an apparent IC₅₀ of 4 ppm. Suppression was observed even at acrolein levels well above those necessary to produce a maximal response. Cigarette smoke, at exposure levels of 10 mg/m³ or higher, caused an immediate and marked sensory irritation response in mice. This response was significantly suppressed by L-menthol even at smoke concentrations as high as 300 mg/m³. Counterirritation by L-menthol was abolished by treatment with a selective inhibitor of Transient Receptor Potential Melastatin 8 (TRPM8), the neuronal cold/menthol receptor. Inclusion of menthol in the cigarette smoke resulted in roughly a 1.5-fold increase in plasma cotinine levels over those observed in mice exposed to smoke without added menthol. These findings document that, L-menthol, through TRPM8, is a strong suppressor of respiratory irritation responses, even during highly noxious exposures to cigarette smoke or smoke irritants, and increases blood cotinine. Therefore, L-menthol, as a cigarette additive, may promote smoking initiation and nicotine addiction.

## Introduction

While the overall rate of cigarette smoking has decreased in the United States and other markets, the proportion of smokers consuming mentholated cigarettes has steadily increased [1]. The rate of menthol cigarette smokers is especially high among beginning smokers, with >50% of initiating smokers reporting the use of menthol cigarettes [1–3]. Recent studies also linked menthol cigarette use to increased frequency of smoking cigarettes, higher incidence of smoking-induced morbidities, increased difficulty to quit smoking and increased use of recreational drugs [2–8].

Menthol, through its pharmacological effects, may be associated with increased smoking initiation, however, the mechanisms underlying this association are not known. Cigarette smoke is an irritant; inhaled irritants stimulate respiratory chemosensory nerves in man, resulting in a variety of responses including burning sensations and cough. In mice, the primary response is a change in breathing pattern, termed braking, which is characterized by a cessation of early expiratory airflow due to glottal closure [9,10]. This braking leads to a diminished breathing frequency which forms the basis for the murine sensory irritation bioassay [11]. Although the mouse mounts compensatory responses (e.g. bradycardia, etc.), the maximal physiological response is a reduction in breathing frequency to 20–30% of control [9]. Sensory nerve stimulation also induces multiple tissue responses, including neurogenic edema and mucous hypersecretion [12–14]. Respiratory chemosensory responses are thought to be protective either by causing noxious sensations (e.g. burning, cough) and initiating avoidance behavior, and/or by altering the rate of absorption of airborne materials into airway epithelium or the bloodstream. Therefore, a suppression of chemosensory responses may facilitate the initiation of smoking behavior by diminishing noxious responses to cigarette smoke and may facilitate addiction to cigarette smoke by enhancing absorption of the addictive smoke constituent, nicotine. The current study was designed to examine the hypothesis that menthol modulates the irritant response and nicotine absorption during first ever exposure to cigarette smoke. Since the effects of menthol on first ever smoking cannot be examined in humans these studies relied on a well characterized mouse model [10,15].

Natural mint plant extracts contain several menthol isomers, of which L-menthol carries the characteristic minty smell and cooling sensory properties. L-Menthol, produced synthetically or purified from natural material, is also the isomer added to menthol cigarettes by the tobacco industry [16]. Menthol acts on the transient receptor potential melastatin 8 (TRPM8) receptor in peripheral sensory neurons, with L-menthol the most potent menthol isomer [17–19]. Our previous studies, relying on a mouse model, have shown that vaporized racemic menthol (a mixture of L-menthol and D-menthol) acts as a counterirritant, attenuating irritant responses to low concentrations of individual tobacco smoke irritants such as acrolein, acetic acid and cyclohexanone [15]. Specific irritant receptors are responsible for activation of respiratory chemosensory nerves [14]. Two important receptor classes are the transient receptor potential ankyrin 1 (TRPA1) receptor activated by acrolein and the transient receptor potential vanilloid 1 (TRPV1) receptor activated by cyclohexanone [15,20–24].

The current study was designed to extend our earlier findings, focusing on the actions of L-menthol, the menthol isomer added to cigarettes, on the murine respiratory irritant response to individual smoke irritants and to cigarette smoke, and on a critical marker of nicotine exposure, cotinine. To be plausibly related to modulation of cigarette smoke-induced responses, L-menthol must be potent and efficacious even in the presence of high levels of irritant. We therefore examined the effects of L-menthol against supra-maximal response levels of the cigarette smoke irritant, acrolein. The role of TRPM8 receptor pathways was assessed by studying the actions of another TRPM8 agonist, eucalyptol, and by examining the effects of the potent TRPM8 antagonist, AMG2850, on any observed effects [15,25,26].

Documentation of a counterirritation effect of L-menthol on individual constituents of cigarette smoke does not provide direct evidence of effects against smoke itself. The effects of L-menthol on cigarette smoke, therefore, were examined using side stream smoke as the inhaled irritant. Since the composition of smoke derived from mentholated versus non-mentholated smoke may differ, we generated smoke from non-mentholated cigarettes (Kentucky Reference 2R4), and added menthol vapor directly to the smoky atmosphere. The average level of menthol in smoke of mentholated cigarettes is estimated to be 8 μmol/l [27]; in the current study the maximal menthol concentration used was 60 ppm (2.4 μmol/l). We examined the effects of L-menthol on the irritant response to a wide range of cigarette smoke concentrations. The effect of AMG2850 was examined to assess the role of TRPM8 in any observed effects of L-menthol on cigarette smoke-induced irritant responses. Finally, we examined blood levels of the primary nicotine metabolite, cotinine, in animals exposed to cigarette smoke with or without added menthol.

## Materials and Methods

### Animals and exposures

All protocols were approved by the Institutional Animal Care and Use Committees of the University of Connecticut (A12-013), Yale University (10928–2011) and Duke University (A067-14-03). Experiments were performed on female C57Bl/6J mice obtained from Jackson Laboratories (Bar Harbor, ME). Each mouse was only exposed once, every exposure was the first ever exposure of that mouse to the test atmosphere. Mice were 8–14 weeks of age at the time of use. Mice were held in a double plethysmograph (Buxco, Inc, Sharon CT) that was connected to a directed airflow nose-only inhalation chamber (CH Technologies, Westwood, NJ) for irritant exposure to allow for continuous monitoring of breathing pattern during the exposure. Mice were lightly restrained in the plethysmograph with a latex collar, at any time they could remove their heads from the exposure atmosphere. This, however, only occurred very rarely (<1/50). If this occurred the exposure was stopped and the mouse excluded from the study. Exposures commenced after a 10 minute baseline period. Exposures to individual irritants were of 15 minute duration, sufficiently long for the maximal response to be induced. Cigarette smoke exposures were typically of 8 minute duration, somewhat less than the duration of a single cigarette burn. Multiple cigarettes were used for exposures of longer duration. Mice were anesthetized immediately after exposure with urethane (1.3 g/kg) followed by exsanguination. When collected, blood was obtained by cardiac puncture with a heparinized syringe from anesthetized mice and then spun at 1000 x g to obtain plasma. When administered, AMG2850 was given at a dose of 15 or 30 mg/kg i.p. The drug was dissolved in solutol (with sonication) at a concentration of 20 mg/ml and then diluted to 3 mg/ml in saline and administered 10 min prior to exposure. Control animals for the AMG2850 studies received vehicle injection.

### Exposure atmosphere generation and characterization

Mice were continuously exposed to constant levels of irritant to allow for the most precise estimation of any irritant-induced changes in breathing pattern. Menthol vapor was generated by passing air through crystalline L-menthol at room temperature. Menthol concentrations ranged between 8–60 ppm, depending on the study. Acrolein and cyclohexanone exposure atmospheres were generated by flash evaporation as described previously [15]. Acrolein irritation was examined at a concentration range from 3–10 ppm. In most studies the target concentration of acrolein was 3 ppm, but there was some day-to-day variation; actual exposure levels for each study are provided below. Cyclohexanone was used at a target concentration of 1500 ppm, with actual exposure levels being provided below. Irritant was mixed with clean- or menthol-laden air prior to entering the nose-only inhalation chamber. To achieve continuous constant concentration smoke exposures, side stream cigarette smoke was continuously generated with a CH Technologies (Westwood, NJ) single cigarette smoking machine. Smoke was generated from Kentucky 2R4 reference cigarettes that had been stored for at least 24 hr at 55% relative humidity. Differing smoke concentrations were obtained by mixing the generator output with differing flow rates of diluting- or menthol-laden air. Generating the atmospheres in this manner ensured that the only difference between the smoke or smoke + menthol atmospheres was the presence of menthol. (The constituents of commercial mentholated and non-mentholated cigarettes differ, which would introduce confounding factors in experiment approaches that relied on these products.)

Airborne acrolein, menthol and cyclohexanone concentrations were determined by gas chromatrography as described previously [15]. For smoke exposures, particulate levels were measured by a Microdust Pro Analyzer (Casella, Buffalo, NY) for concentrations below 50 mg/m^3^. For concentrations above 50 mg/m^3^, nose-only chamber air samples were drawn through a 0.45 μm pore filter, with particulate mass determined gravimetrically. Airborne carbon monoxide levels were monitored continuously during exposure with a General DCO1001 meter (General Specialty Tools & Instruments, New York, NY). For airborne nicotine determination, air samples were drawn from the nose only chamber through a 0.45 μm filter followed by two midget impingers each containing 5 ml of HPLC mobile phase (see below). The midget impinger fluids were mixed and the filter placed in the fluid for dissolution of nicotine. This allowed for determination of total nicotine content irrespective of whether it was in the particulate or vapor phase. Nicotine levels in the fluid were assessed by HPLC as described by Hariharan and vanNoord using a 25 cm Supelco Discovery C18 column (Supelco, Inc., Bellefonte, PA), UV detection (Shimadzu SPD-20A uv-vis detector, Shimadzu, Columbia, MD) and a mobile phase of 91:9 phosphate-citrate buffer: acetonitrile containing triethylamine and heptane sulfonate [28].

### Breathing pattern analysis

Nasal chemosensory nerve stimulation results in the sensory irritant response, characterized by a prolonged braking (slowing or cessation of flow) at the onset of each expiration [9,10]. As in our previous study, breathing patterns were monitored continuously during the pre-exposure acclimatization period and exposure period using EMKA software (EMKA Technologies, Inc., Falls Church, VA). This software provided measures of breathing frequency, tidal volume, duration of braking during early expiration, peak inspiratory and peak expiratory flow. Data collected from each breath were averaged to provide running 1 minute averages. Irritant exposure caused a typical prolonged braking at the start of each expiration. The duration of this braking period was quantified as the duration required to achieve 25% of the peak expiratory flow rate for each breath [15]. Data are presented either as a time course (running 1 min averages) or the average response over the entire exposure.

### Determination of cotinine in mouse blood

Plasma samples were frozen at -20°C for later analysis. Cotinine levels in plasma were measured in duplicate by ELISA (Calbiotech, Spring Valley, CA) following the manufacturer’s recommendations.

### Statistical Analysis

Data are reported as mean ± SEM. Statistically outlying data points (deviant from the mean by greater than 3x the SD of the group) were excluded prior to statistical analysis. Data were analyzed by XLSTAT software (Addinsoft, New York, NY). Data were compared by ANOVA followed, as appropriate, by Newman-Keuls test. Analysis of time dependent changes was performed by repeated-measures ANOVA. Log-linear regression was performed to estimate potency from the concentration response experiments. When necessary, data were corrected for heteroscedasticity by log transformation. A p-value less than 0.05 was required for statistical significance.

## Results

### Effect of L-menthol on individual irritants

**Fig 1:**
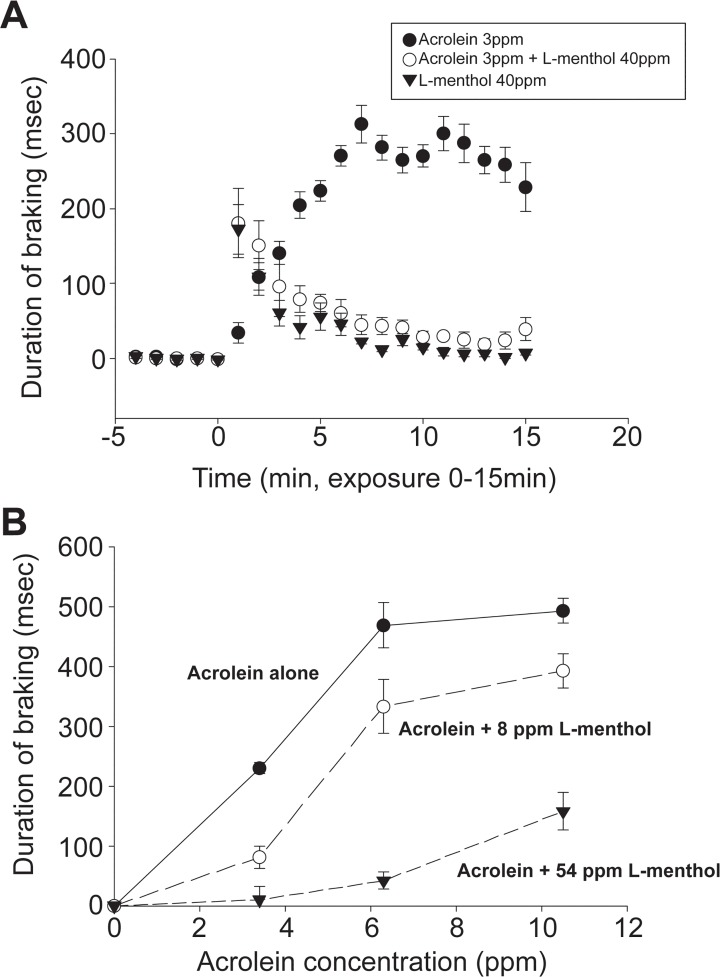
Effect of L-menthol on the murine respiratory irritation response to acrolein. (*A*) Change in duration of braking (DB) during exposure to 3 ppm acrolein, 40 ppm L-menthol vapor or the combination. Data are presented as the 1 minute average DB level during the baseline (-5 to 0 minute) or the exposure (0 to 15 minute) period, expressed as mean ± SEM (n = 4–7 mice per group). Repeated measures ANOVA followed by Newman-Keuls test indicated the response to the combination was significantly lower than that to acrolein alone (p<0.001). At all exposure times, the response to acrolein+L-menthol was virtually identical to that of L-menthol alone.(*B*) Effect of 8 or 54 ppm L-menthol on responses to 3, 7 or 11 ppm acrolein. Data are presented as the average DB during the 15 minute exposure (corrected for baseline), expressed as mean ± SEM (n = 5–7 per group). The responses in both the 8 and 54 ppm L-menthol groups were significantly lower than for acrolein alone, and differed significantly from each other (p<0.0001, two-factor ANOVA).

At an exposure concentration of 3 ppm, acrolein produces an immediate and marked sensory irritant response (Fig. 1A). L-menthol alone (40 ppm) produced a transient irritant response; at this concentration. L-menthol blocked the sensory irritant response to 3 ppm acrolein, indicating that it was an effective counterirritant (Fig. 1A). This counterirritant effect was observed throughout the 15 minute acrolein exposure. L-Menthol was also effective at suppressing the irritant response to higher concentrations of acrolein (Fig. 1B). The maximal irritant response to acrolein alone was observed at an exposure concentration of 6 ppm. At this concentration breathing frequency was reduced to less than 30% of baseline, which represents the physiologically maximal response level [9]. Thus, the concentration range of acrolein spanned the entire response range (from minimal to maximal) of the sensory irritant response. L-menthol was an effective counterirritant even at acrolein exposure levels exceeding 10 ppm. An apparent concentration response relationship was observed for L-menthol-induced counterirritation, with greater irritant response suppression being observed at 54 than 8 ppm L-menthol at all exposure concentrations of acrolein (Fig. 1B).

**Fig 2:**
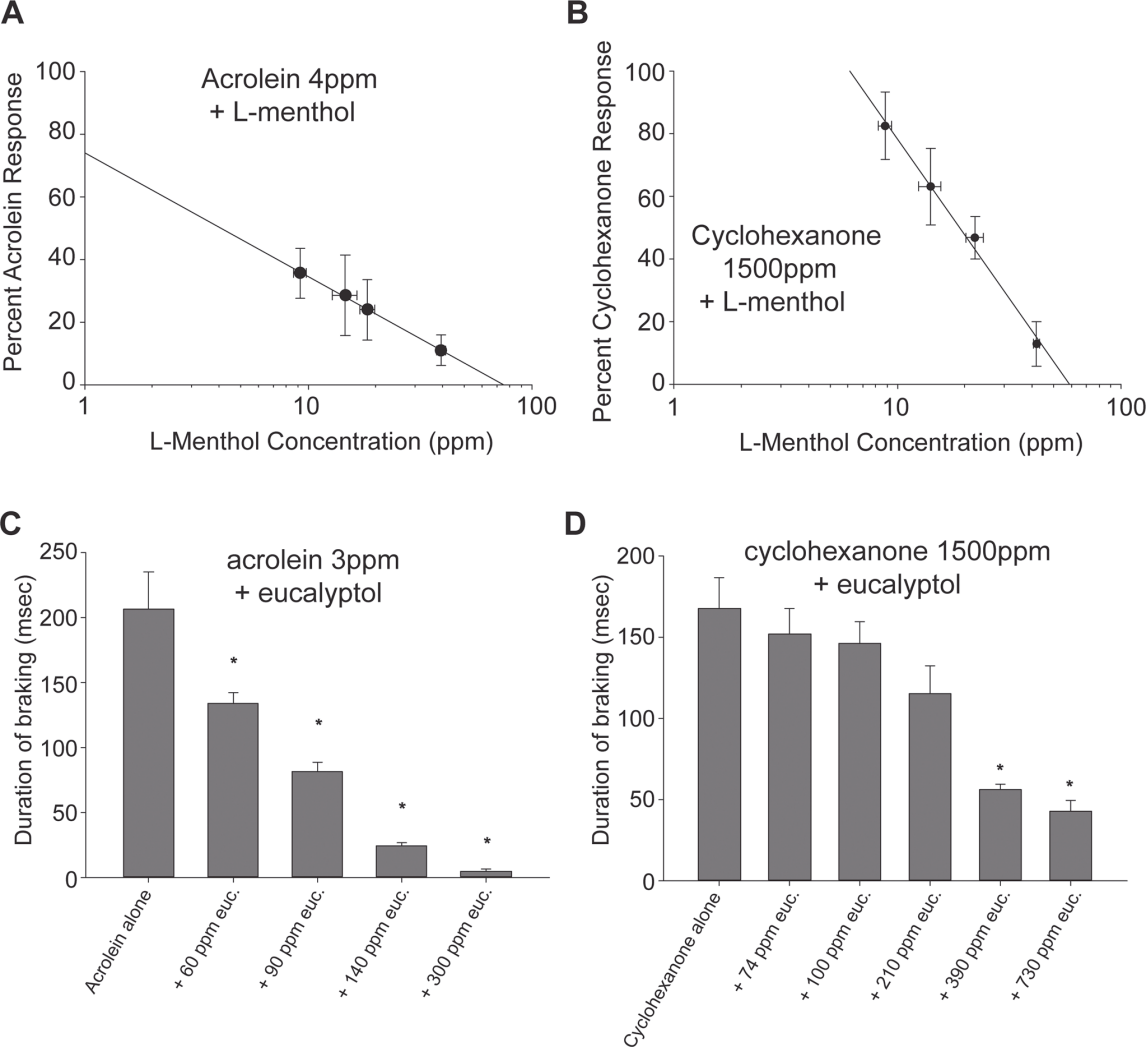
Dose-response relationships of effects of TRPM8 agonists (L-menthol, eucalyptol) on respiratory irritation by acrolein or cyclohexanone in mice. (*A*) Logarithmic concentration response curve for the effect of L-menthol (9, 14, 18 or 39 ppm) on acrolein (4 ppm)-induced irritation. Data are presented as the average DB level during the 15 minute exposure (corrected for baseline), expressed as mean ± SEM (n = 5–7 mice per group). Shown is the log linear plot of the response (percent of control) in the acrolein-L-menthol groups. The response to acrolein was attenuated at all L-menthol exposure concentrations (p<0.0001 ANOVA followed by Newman-Keuls test). A significant correlation (r^2^ = 0.99, p<0.01) was observed, with an apparent IC_50_ of 4 ppm (95% confidence limits, 3.3–4.9 ppm). (*B*). Logarithmic concentration response curve for the effect of L-menthol (8, 18. 24 pr 40 ppm) on cyclohexanone (1500 ppm)-induced irritation. Data are presented as the average DB level during the 15 minute exposure (corrected for baseline), expressed as mean ± SEM (n = 5–8 mice per group). Shown is the log linear plot of the response (percent of control) in the cyclohexanone-L-menthol groups. The response to cyclohexanone was significantly attenuated by L-menthol in the 24 and 40 ppm L-menthol groups (p<0.05, ANOVA followed by Newman-Keuls test). A significant correlation (r2 = 0.99, p<0.01) was observed, with an apparent IC_50_ of 19 ppm (95% confidence limits, 16–24 ppm). This IC_50_ is higher than that observed for attenuation of the irritant response to acrolein (Fig. 1C). (*C*) Effect of eucalyptol on the irritation response to acrolein. Data are presented as the average DB level during the 15 minute exposure (corrected for baseline) expressed as mean ± SEM (n = 5–8 mice per group). The response to 3 ppm acrolein was attenuated by eucalyptol (p<0.0001, ANOVA) with the responses in the 60, 90, 140 and 300 ppm groups all being significantly lower than the response to acrolein alone (* p<0.05 compared to cyclohexanone alone, Newman-Keuls test). Log linear regression analysis of the response (percent of control) in the acrolein-eucalyptol groups revealed a significant correlation (r2 = 0.99, p<0.01) with an apparent IC_50_ of 73 ppm (95% confidence limits, 69–80 ppm). (*D*) Effect of eucalyptol on the irritation response to cyclohexanone. Data are presented as the average DB level during the 15 minute exposure (corrected for baseline) expressed as mean ± SEM (n = 5–8 mice per group). The response to 1500 ppm cyclohexanone was attenuated by eucalyptol (p<0.0001, ANOVA) with the response in the 390 and 730 ppm being significantly lower than cyclohexanone alone (*, p<0.05 compared to cyclohexanone alone, Newman-Keuls test). Log-linear regression analysis of the response (percent of control) in the cyclohexanone-eucalyptol groups revealed a significant correlation (r2 = 0.96, p<0.01), with an apparent IC_50_ of 305 ppm (95% confidence limits, 220–470 ppm). This IC_50_ value is higher than the IC_50_ observed for eucalyptol attenuation of acrolein-induced irritation (Fig. 1C).

**Fig 3:**
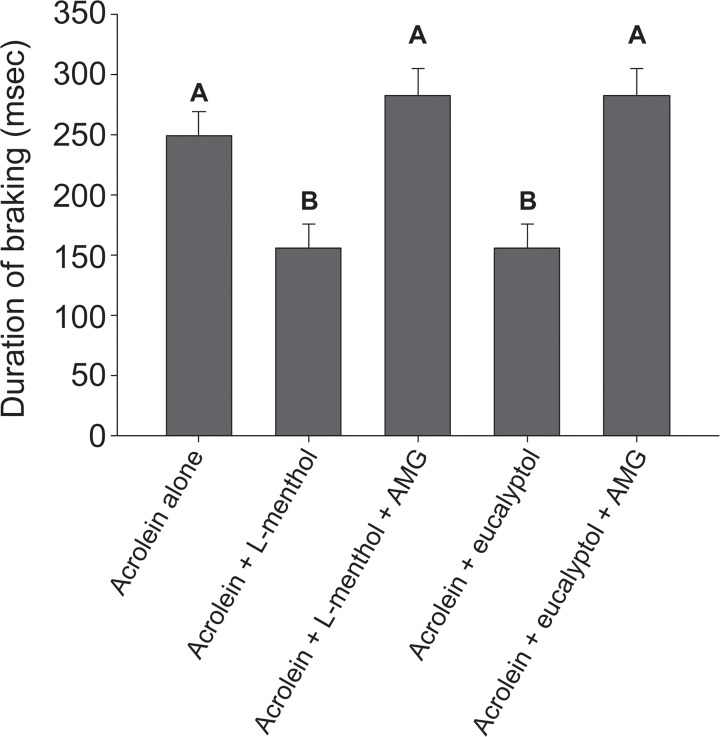
Effect of TRPM8 inhibitor, AMG2850 (15 mg/kg), on inhibition of 3 ppm acrolein response by 7 ppm L-menthol or 800 ppm eucalyptol. Data are presented as the average DB level during the 15 minute exposure (corrected for baseline), expressed as mean ± SEM (n = 3–5 mice per group). Data were analyzed by ANOVA (p<0.001) followed by Newman-Keuls test; bars with differing superscripts differ from each other at the p<0.05 level. The counterirritant effects of L-menthol were absent in AMG2850-pretreated mice.

L-menthol suppressed acrolein (4 ppm)-induced irritation with an apparent IC_50_ of 4 ppm (95% CL: 3.3–4.9 ppm, Fig. 2A). The counterirritant effects of L-menthol were not specific to acrolein, but were also apparent against the TRPV1 agonist cyclohexanone. L-menthol suppressed the cyclohexanone-induced irritant response with an apparent IC_50_ of 19 ppm (95% CL: 16–24 ppm, Fig. 2B). Thus, although an effective counterirritant, L-menthol is less potent a counterirritant against cyclohexanone than acrolein. The antinociceptive effects of L-menthol are thought to be mediated via stimulation of the TRPM8 receptor. We next used two approaches to provide information on the role of TRPM8 in the counterirritant effect of L-menthol. First we examined the counterirritant effects of eucalyptol, another TRPM8 agonist (Fig. 2C & D). Eucalyptol produced concentration dependent suppression of the irritant responses to both acrolein (apparent IC_50_ of 73 ppm, 95% CL: 69–80 ppm) and cyclohexanone (apparent IC_50_ of 305 ppm, 95% CL: 220–470 ppm). As for L-menthol, eucalyptol was a less potent counterirritant against cyclohexanone than acrolein (Fig. 3). Second, we examined the effect of the TRPM8 antagonist AMG2850 on the counterirritant effects of both L-menthol and eucalyptol against the irritant acrolein (Fig. 3). For both L-menthol and eucalyptol, the counterirritant effects were abolished by the TRPM8 competitive antagonist AMG2850 (15 mg/kg). Taken together, these data show that L-menthol, the menthol isomer added to mentholated cigarettes, is a potent and efficacious counterirritant.

### Effect of L-menthol on cigarette smoke

**Fig 4:**
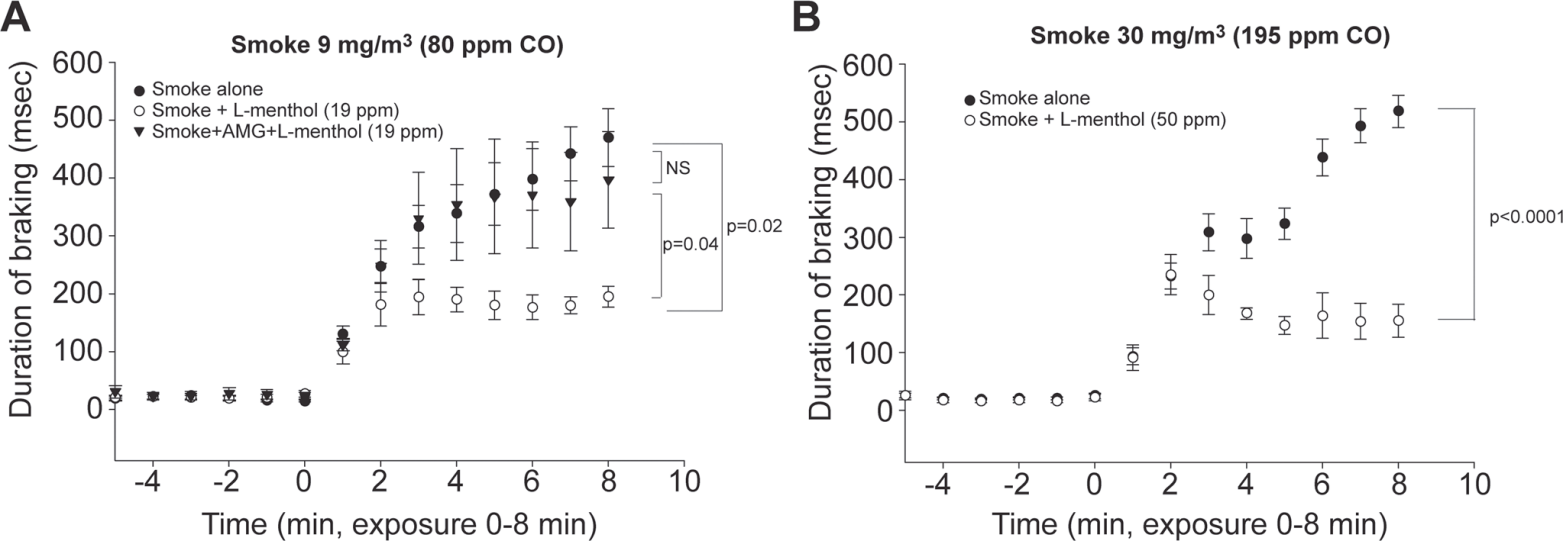
Effect of L-menthol on the murine respiratory irritation response to cigarette smoke. Time course of response to smoke alone and smoke + L-menthol and smoke + L-menthol in AMG2850-pretreated mice (30 mg/kg). Data are presented as the 1 minute average DB level during the baseline (-5 to 0 minute) or the exposure (0 to 8 minute) period. AMG2850 did not modulate the response to menthol alone (data not shown.). (A) At a smoke concentration of 9 mg/m^3^ (80 ppm carbon monoxide) and L-menthol concentration of 19 ppm, the response to smoke was significantly attenuated by L-menthol, and the attenuation was blocked by AMG2850 (repeated measures ANOVA, p<0.001, Newman-Keuls; p values indicated in the figure, n = 4–12 mice per group). (*B*) At a smoke concentration of 30 mg/m^3^ (195 ppm carbon monoxide) and L-menthol concentration of 50 ppm, the response was significantly attenuated by L-menthol (repeated measures ANOVA; p value indicated in the figure, n = 4–6 mice per group).

**Fig 5:**
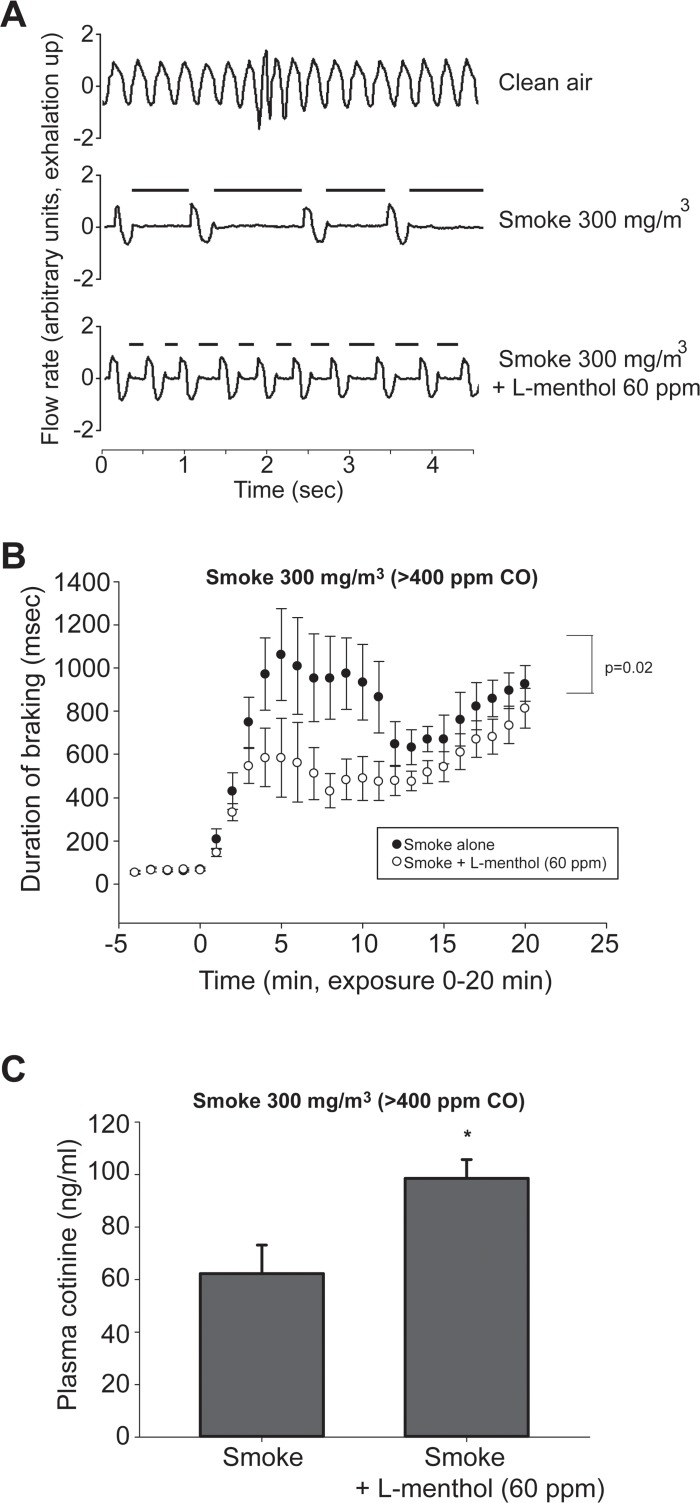
Effect of L-menthol on murine ventilation response to high concentration of cigarette smoke and on serum cotinine levels. (*A*) Breathing patterns of a mouse at baseline (top), during exposure to 300 mg/m^3^ (>400 ppm carbon monoxide) side stream cigarette smoke (middle), and side stream smoke combined with 60 ppm L-menthol vapor (bottom). Shown are representative recordings of respiratory flow rate (inspiration downward, expiration upward). For the sake of clarity, the recordings are representative of minutes 6–9 of exposure when the effects of smoke and menthol were maximal and at equilibrium, see Fig. 5B. The prolonged braking at the onset of expiration during smoke exposure is readily apparent and is indicated by the heavy bars. This effect is measured as the duration of braking (DB) during each expiration. (*B*) Time course of the response to 300 mg/m^3^ smoke (>400 ppm carbon monoxide). Data are presented as the 1 minute average DB level during the baseline (-5 to 0 minute) or the exposure (0 to 20 minute) period, expressed a mean ± SEM (n = 7–8 mice per group). The response was significantly attenuated by 60 ppm L-menthol (repeated measures ANOVA, p value provided in the figure), particularly at the start of exposure. (*C*) Cotinine levels in blood drawn immediately after 20 minute exposure to 300 mg/m^3^ smoke or 300 mg/m^3^ smoke +60 ppm L-menthol. Data are expressed as mean ± SEM (n = 7–8 mice per group). These were the same mice whose breathing response is shown in (*B*) above. The two groups differed from each other (*) at p = 0.015 level (t-test). The airborne nicotine levels averaged 30 mg/m^3^ and were identical in the smoke-alone and smoke + L-menthol groups. Cotinine levels in control (non-exposed) mice averaged less than 1 ng/ml.

Having demonstrated L-menthol was a potent and efficacious counterirritant we next examined the effect of L-menthol on cigarette smoke-induced irritation. Cigarette smoke, at relatively low concentration (9 mg/m^3^) produced an immediate and marked sensory irritation response (Fig. 4A). The irritant response was suppressed by approximately 50% by inclusion of 19 ppm L-menthol. The suppression of the cigarette smoke irritant response by L-menthol did not occur in animals pretreated with the TRPM8 antagonist AMG2850 (Fig. 4A). (Because we used a higher concentration of L-menthol in this study than in the acrolein/eucalyptol study, Fig. 3, the competitive antagonist AMG2850 was administered at a dose of 30 mg/kg rather than 15 mg/kg). As observed for acrolein, L-menthol exhibited counterirritant effects against a wide range of cigarette smoke exposure concentrations. The irritant response to a moderate concentration of cigarette smoke (30 mg/m^3^) was suppressed by approximately 50% by a menthol concentration of 50 ppm (Fig. 4B). Even at a high cigarette smoke concentration of cigarette smoke (300 mg/m^3^) the initial irritant response was also suppressed by approximately 50% by 60 ppm L-menthol (Fig. 5A, B). At this concentration, cigarette smoke reduced breathing frequency to less than 30% of control; this is a physiologically maximal response level [9]. The exposure to 300 mg/m^3^ cigarette smoke was continued for 20 minutes, after which time mice were euthanized and blood drawn for determination of cotinine levels. L-menthol continued to exert counterirritant effects throughout the 20 minute exposure, but these effects were diminished at the end, compared to the beginning of the smoke exposure (Fig. 5B). The airborne nicotine concentration during the exposure was 30 mg/m^3^ and was identical in the smoke alone and smoke plus menthol groups. The 20 minute exposure resulted in increased blood cotinine levels to greater than 50 ng/ml compared to <1 ng/ml in control (non-smoke exposed mice). Blood cotinine levels were approximately 1.5 fold higher in animals exposed to smoke plus L-menthol than in animals exposed to smoke alone (p<0.05, Fig. 5C).

An increase in blood cotinine levels by inclusion of menthol vapor in the cigarette smoke might be attributable to increased smoke inhalation and increased delivered dosage of nicotine. A detailed analysis of the breathing patterns during 300 mg/m^3^ smoke exposure with and without L-menthol vapor is provided in Table 1. Breathing frequency was diminished to less than one-third of baseline control during smoke exposure; breathing frequency was somewhat higher in smoke plus L-menthol than in smoke-alone exposed mice, but the difference did not achieve statistical significance (p = 0.065). Minute ventilation was decreased to approximately one-half of baseline values and was virtually identical in smoke-alone and smoke plus L-menthol exposed mice, indicating that L-menthol did not increase the inhaled burden of nicotine. Compared to smoke alone, smoke+ L-menthol mice exhibited a decreased peak inspiratory flow and decreased peak expiratory flow, suggesting that L-menthol slowed the active phases of inspiration and expiration. Thus, while the duration of braking was attenuated by L-menthol, an elongation of the active phases (e.g. non breath-hold) of respiration occurred resulting in little net change in the minute ventilation.

**Table 1:** Breathing patterns in mice exposed to smoke, menthol or the combination ^1^

	Breathing Frequency (breaths/min)	Minute Ventilation (ml/min)	Peak Inspiratory Flow normalized to tidal vol. (ml/sec per ml)	Peak Expiratory Flow Normalized to tidal vol. ml/sec per ml
Baseline	273^A^±17	48^A^±5	16^A^±1.5	14^B^±0.9
Smoke-alone	76^C^±16	21^B^±7	14^B^±1.7	16^A^±1.0
Smoke+menthol	95^C^±19	23^B^±5	11^C^±1.8	13^B^±1.4
Menthol-Alone	241^B^±29	47^A^±5	15^A^ ^,^ ^B^±3.1	14^B^±0.8

1 Average values throughout the exposure are shown and are presented as mean ± SEM. These are the same mice reported in Fig. 5A and 5B. Each group contained 5–8 mice. Mice were exposed to cigarette smoke (300 mg/m^3^, >400 ppm carbon monoxide) for 20 minutes with and without 60 ppm L-menthol. Data were analyzed by ANOVA followed by Newman-Keuls test; groups with differing superscripts differed at the p<0.05 level.

^A,B,C^ Data were analyzed by ANOVA followed by Newman Keuls test. Groups with differing superscripts differed at the p<0.05 level.

## Discussion

The current results document that L-menthol is a highly efficacious counterirritant that suppresses chemosensory irritant responses during exposures to very high concentrations of individual irritants. L-menthol diminished responses to high level exosures to acrolein, an irritant that acts via stimulation of the TRPA1 receptor, and to cyclohexanone an irritant that acts via stimulation of the TRPV1 receptor. These findings complement the results of our previous study in which mice were exposed to intermediate, non-saturating, irritant levels [15]. We also observed that L-menthol suppressed the irritant response to cigarette smoke. This outcome could not be predicted from our previous work since cigarette smoke contains hundreds of additional irritants, including nicotine and reactive particulates, that activate diverse chemosensory systems and irritant receptors. It has been unknown whether these mechanisms are also sensitive to inhibition by L-menthol.

The counterirritant effects of L-menthol are likely due to stimulation of the TRPM8 receptor. The current results indicate that two TRPM8 agonist vapors, L-menthol and eucalyptol, are both counterirritants, and the counterirritant effects of both are blocked by AMG2850, a newly developed highly specific TRPM8 antagonist which we and others have validated *in vitro* and *in vivo* in previous studies [25,26,29]. In this regard, the effects of L-menthol on respiratory chemosensory nerve responses may be similar to its actions on responses mediated by sensory nerves innervating other organ systems. For example, L-menthol acts as an analgesic of acute, inflammatory and neuropathic pain mediated by nociceptors, the sensory neurons signaling pain [29–31]. Similar to the sensory nerves innervating the airways, nociceptors derived from dorsal root ganglia express TRPA1 and TRPV1 receptors. When activated, these receptors initiate the sensation of pain [20,32,33]. L-menthol, in a TRPM8-dependent manner, suppressed pain behavior in mice when these receptors were activated [29]. TRPM8 is expressed in a mostly separate population of cold-sensitive sensory fibers that may suppress input from respiratory chemosensory neurons and nociceptors by engaging inhibitory neuronal circuits in the trigeminal nucleus and spinal cord.

The potency of L-menthol in suppressing the irritant response to acrolein and cyclohexanone differed, with greater potency being observed towards the TRPA1 agonist, acrolein [20]. Eucalyptol also demonstrated greater potency against acrolein- than cyclohexanone-induced irritation suggesting that heightened potency against TRPA1 compared to TRPV1 agonists is a generalized response pattern for TRPM8 agonists. TRPV1 is expressed more widely in sensory ganglia than TRPA1 that is co-expressed with TRPV1 in a subset of neurons [33,34]. L-menthol may have differential effects on inputs from these co-expressing neurons, while leaving input from other TRPV1-expressing neurons unaffected.

The current results demonstrate that L-menthol effectively suppresses the irritant response to even high concentrations of cigarette smoke. Because we added L-menthol directly to freshly generated smoke, the suppression is clearly due to L-menthol itself, and not due to modulation of smoke constituents due to inclusion of menthol in the burning cigarette. As observed for the individual irritants, the counterirritant effect of L-menthol against cigarette smoke was blocked by the TRPM8 antagonist AMG2850, supporting a role for TRPM8 receptor pathways in this effect. The maximal L-menthol concentration used in the current study, 60 ppm (2.4 μmol/l), is less than the concentration reported for mentholated cigarette smoke (8 μmol/l), indicating there is more than sufficient L-menthol present in mentholated cigarette smoke to exert pharmacological counterirritant effects, were man to be similarly responsive to the mouse [27,35,36]

L-Menthol vapor produced multiple changes in breathing patterns in mice. At the exposure level of 30 mg/m^3^, the mice exhibited the maximal physiological response of greater than 70% reduction in breathing frequency. Although the mice did not attempt to withdraw from the exposure they were clearly experiencing physiological stress at the response level [9,11]. Even at this maximal response level, menthol was an effective counterirritant. It diminished the duration of braking, whether induced by individual irritants (Figs. 1 and 2) or cigarette smoke (Figs. 4 and 5).

In the mouse the expiratory braking response is due to glottal closure [9,10]. When the glottis opens, air is forced out with great force, due to the pressure accumulated during expiratory muscle contraction against the closed glottis [10]. By diminishing the duration of braking, L-menthol would be expected to lead to diminished pressure accumulation and diminished expiratory flow when the glottis opens. This was observed in the cigarette smoke plus L-menthol compared to cigarette-smoke alone exposed mice (Table 1). In addition, L-menthol diminished inspiratory flow rates, which would lead to prolonged times of inspiration. The net effect of the diminished duration of braking with prolonged active expiration and inspiration was that there was no difference in the minute ventilation between cigarette smoke alone or cigarette smoke plus menthol exposed mice. That minute ventilation was similar indicates the inhaled burden of nicotine is similar in both smoke alone and smoke+menthol exposed mice.

Cotinine is the major metabolite of nicotine [37]. In our present study, using naïve mice mimicking novice smokers never exposed to smoke or menthol before, blood cotinine levels were higher in animals exposed to cigarette smoke plus L-menthol than in animals exposed to cigarette smoke alone. Blood cotinine concentrations were determined after the end of exposure when blood cotinine levels in mice and humans are known to reach equilibrium and closely correlate with blood nicotine levels [38–40]. The mechanisms through which menthol increased blood cotinine levels in our experiments are not known. The effect of L-menthol is not due to changes in inspired nicotine burden (see above). It is not likely that L-menthol directly enhanced conversion of nicotine to cotinine (e.g. by inducing the enzymes responsible) due to the short time course of the experiment. Thus, this effect is most likely due to enhanced systemic delivery of nicotine with subsequent conversion to cotinine. Although speculative, it is possible that initiation of chemosensory responses such as mucous hypersecretion by cigarette smoke might serve to retard nicotine absorption; if so, blockade of these responses by L-menthol would accelerate nicotine delivery to the bloodstream. Further studies would be needed to confirm this possibility.

The current study relied on addition of L-menthol vapor to cigarette smoke generated from non-mentholated cigarettes to ensure the only difference in exposure was the presence of L-menthol. To our knowledge, no other study has shown L-menthol-induced elevation in blood cotinine levels in first time exposure to cigarette smoke in a paradigm that indicated the effect must be due to alterations in nicotine disposition rather than changes in inhaled burden.

The current studies were performed in first ever exposed mice. Therefore, these results may be particularly relevant in predicting the effects of menthol in cigarettes in novice smokers. By blocking noxious chemosensory responses L-menthol may render cigarette smoke more easily tolerated by first time smokers.

Menthol’s effects may differ in chronic smokers in which sensory irritation responses may be altered and neuronal and pulmonary remodeling and inflammation have manifested [41–44]. In a clinical study in long-term smokers with ∼17 years of smoking history, smoking of mentholated cigarettes was shown to slow the metabolic clearance of intravenously injected deuterated nicotine, suggesting that menthol may affect nicotine metabolism and increase systemic nicotine exposure [45]. The extended presence of nicotine may underlie the observed differences in smoking frequency and the increased difficulty of menthol smokers to quit [8,45–47]. It remains unclear whether these effects are mediated by TRPM8, or through other mechanisms sensitive to menthol.

Mice and humans differ in their respiratory system anatomies and developed different respiratory defense mechanisms to protect from irritant damage. Mice are obligate nasal breathers and smoke irritants first interact with trigeminal nerve endings in the nose followed by interactions with vagal nerves in the lower airways. The murine sensory irritation response, resulting in diminished respiratory rates, is predominantly triggered by trigeminal nerves, and irritants are scavenged (scrubbed) effectively while passing through the nose [9,48]. In contrast to mice humans can inhale air through both nose and mouth. Depending on the inhalation route, irritants activate respiratory defensive reflexes through either trigeminal (sneezing) or vagal neurons (cough), or both systems. The initiating human smoker inhales smoke through the mouth, activating cough-inducing vagal neurons innervating the larynx, and also trigeminal neurons innervating the mouth and nasal passages, exposed during exhaling or retronasal passage of smoke. While these physiological species differences are evident, the mechanisms of chemical sensing and the effects of menthol are highly similar suggesting that data gathered from mice closely reflect human physiology. Menthol suppresses irritation responses and has analgesic effects in both species, and human and murine TRPM8 channels have almost identical sensitivity to menthol, eucalyptol and related cooling agents [17,19,49]. Menthol has anti-tussive effects in irritant-exposed humans either when inhaled through the mouth or when administered via the nose, suggesting that both trigeminal and vagal neurons can mediate its counterirritant effects [35,36,50].

## Conclusions

L-menthol is an additive in 90% of cigarettes, including those not marketed as being mentholated. Its use as a cigarette additive is controversial. The current results provide evidence that L-menthol may be acting as a pharmacological agent in cigarette smoke, acting to both diminish the irritant response and enhance nicotine absorption by limiting chemosensory nerve-mediated irritant effects. Citing primarily epidemiological evidence, the US FDA concluded that addition of menthol to cigarettes may be associated with increased initiation and progression to heavy smoking [51]. The current results provide a plausible mechanistic basis for the association between mentholated cigarettes and enhanced smoking initiation and progression. Menthol may also promote initiation of consumption of novel nicotine delivery products such as electronic cigarettes, with many varieties containing menthol and related aromas with as yet unknown health effects [52].
